# Cultivar-Dependent Phytochemical Composition and Cosmetic Potential of Grapevine Callus Cultures

**DOI:** 10.3390/molecules31203610

**Published:** 2026-10-10

**Authors:** Marta Sharafan, Magdalena A. Malinowska, Arnaud Lanoue, Florent Magot, Nathalie Giglioli-Guivarc’h, Elżbieta Sikora, Agnieszka Szopa

**Affiliations:** 1 Department of Organic Chemistry and Technology, Faculty of Chemical Engineering and Technology, CUT Doctoral School, Cracow University of Technology, 24 Warszawska St., 31-155 Cracow, Poland; marta.sharafan@doktorant.pk.edu.pl; 2 Department of Organic Chemistry and Technology, Faculty of Chemical Engineering and Technology, Cracow University of Technology, 24 Warszawska St., 31-155 Cracow, Poland; magdalena.malinowska@pk.edu.pl; 3 UR 2106 Biomolécules et Biotechnologies Végétales, UFR des Sciences Pharmaceutiques, Université de Tours, 31 av. Monge, F37200 Tours, France; arnaud.lanoue@univ-tours.fr (A.L.); florent.magot@univ-tours.fr (F.M.); nathalie.guivarch@univ-tours.fr (N.G.-G.); 4 Department of Medicinal Plant and Mushroom Biotechnology, Faculty of Pharmacy, Jagiellonian University Medical College, 9 Medyczna St., 30-688 Cracow, Poland; a.szopa@uj.edu.pl

**Keywords:** *Vitis vinifera*, callus-derived extracts, plant cell cultures, stilbenoids, *E*-*ε*-viniferin, resveratrol derivatives, antioxidant activity, tyrosinase inhibition, anti-aging cosmetics, biotechnology-derived cosmetic actives

## Abstract

Plant callus cultures are increasingly explored as a source of skin-care ingredients because they enable the production of biomass rich in bioactive metabolites under controlled and reproducible conditions. Callus cultures offer a promising platform for the controlled production of bioactive metabolites independent of environmental and seasonal variability, making them attractive sources of innovative dermatological raw materials. In the present study, callus cultures of six grapevine (*Vitis vinifera* L.) cultivars (Chardonnay, Hibernal, Regent, Marechal Foch, Seyval Blanc, and Rondo) were established and optimized to produce biologically active extracts with potential cosmetic applications. Cultures were maintained on Murashige and Skoog (MS) and Schenk and Hildebrandt (SH) media supplemented with different combinations of plant growth regulators. Biomass growth was evaluated using the growth index (Gi), while phytochemical profiling was performed using UPLC-MS/MS. A total of twelve metabolites were identified, representing several compound classes, including amino acids, phenolic acid (e.g., gallic acid), flavan-3-ols, and stilbenoids. Distinct cultivar- and medium-dependent differences in metabolite accumulation were noted. The highest total metabolite content was detected in cv. Marechal Foch cultivated on MS medium, reaching 756.85 mg/g DW, while extracts of cvs. Chardonnay and Hibernal grown on SH medium accumulated particularly high levels of stilbenoids. The antioxidant potential of the extracts was assessed using DPPH, ABTS, and FRAP assays, and skin-whitening activity was evaluated through tyrosinase inhibition testing. Seyval Blanc extracts demonstrated the strongest antioxidant potential in the FRAP assay (3.10 mM TE/L), whereas Chardonnay cultured on MS medium exhibited the highest tyrosinase inhibitory activity (65.06%). These findings demonstrate that grapevine callus cultures represent a valuable and sustainable source of antioxidant and skin-whitening compounds and highlight their considerable potential as multifunctional active ingredients for innovative dermatological formulations.

## 1. Introduction

Accelerated skin aging and degeneration are multifactorial biological processes characterized by progressive structural and functional alterations resulting from intrinsic mechanisms, including chronological aging and genetic predisposition, and extrinsic factors such as ultraviolet radiation, pollution and oxidative stress. Among these, chronic UV exposure is recognized as one of the primary causes of premature aging due to the excessive generation of ROS, which disrupts cellular homeostasis and promotes the degradation of extracellular matrix components. The resulting reduction in collagen and elastin content contributes to decreased skin elasticity, wrinkle formation and pigmentation disorders. Consequently, the identification of effective bioactive compounds capable of protecting the skin against oxidative damage remains an important objective in cosmetic and dermatological research [1,2].

Modern phytocosmetology is a rapidly developing field. It provides opportunity to identify and isolate natural substances of plant origin, which could be effectively applied in skin-care formulations. Secondary metabolites obtained from plants such as stilbenoids, flavonoids or phenolic acids are proven to exhibit antioxidant, anti-inflammatory and enzymes-inhibitory properties that may contribute to the prevention of skin aging [3,4]. Plant-derived compounds have attracted considerable interest as potential bioactive ingredients, because of their broad spectrum of biological activities supporting their application in anti-aging formulations. For instance, *Aloë vera* contains aloin A and B, which block the collagenase activity and production of matrix metalloproteinases (MMPs) [5]. *Panax ginseng* contains characteristic saponins—ginsenosides, which have been proved to scavenge free radicals [6]. Flowers of *Hypericum preforatum* inhibit enzymes involved in anti-aging such as collagenase, elastase, hyaluronidase and tyrosinase [7]. Red grape (*Vitis vinifera*) leaf extract also possessed tyrosinase inhibition activity thanks to the presence of *E*-resveratrol [8].

Despite their promising biological activity, the use of conventionally cultivated plants is limited by substantial variability in metabolite composition. Environmental conditions and cultivation practices significantly influence the qualitative and quantitative profiles of secondary metabolites, making it difficult to obtain extracts with consistent phytochemical profile and biological activity. This variability represents a major challenge for the development of standardized ingredients. Plant biotechnology offers an alternative approach by enabling biomass production under controlled in vitro conditions. Plant in vitro cultures provide cultivation systems that are independent of environmental conditions while allowing precise regulation of constant metabolite production and growth conditions [9,10,11,12,13,14].

Among in vitro cultures, callus cultures have attracted particular attention because of their rapid proliferation capacity and ability to synthesize valuable biologically active secondary metabolites [15,16]. Callus-derived biomass is an attractive source of standardized plant material for dermatological applications. Previous studies have reported antioxidant, anti-aging, photoprotective, and skin-brightening properties of extracts obtained from callus cultures [17,18,19,20,21,22,23,24,25,26,27,28,29]. These biological effects have been associated with inhibition of collagenase, elastase, hyaluronidase and tyrosinase activity and enhancement of antioxidant defense mechanisms. However, the reported activity varies considerably depending on the plant species, culture cultivation and extraction procedures. This variability also creates the opportunity to identify species with the greatest potential for secondary metabolite production and subsequently optimize their culture conditions to maximize the biosynthesis and accumulation of target compounds. Therefore, identifying suitable plant species and optimizing culture conditions may represent a promising strategy for obtaining higher and more consistent yields of biologically active secondary metabolites [11,30]. A species of particular interest is *Vitis vinifera* L. (grapevine). It has been extensively investigated as a source of secondary metabolites, particularly phenolic compounds. Moreover, *V. vinifera* comprises many cultivars, which can differ considerably in their phytochemical composition and capacity to produce secondary metabolites. The considerable diversity of cultivars makes *V. vinifera* a promising candidate for the development of an optimized in vitro culture system aimed at enhancing the production of bioactive compounds [31,32,33,34,35,36,37].

Although *V. vinifera* is widely recognized as a valuable source of biologically active phytochemicals, the cosmetic potential of grapevine callus cultures remains largely unexplored. Therefore, the present study was designed to develop and optimize callus cultures of six grapevine cultivars (cvs.), Chardonnay, Hibernal, Regent, Marechal Foch, Seyval Blanc, and Rondo, and to investigate their suitability as novel biotechnology-derived cosmetic raw materials. The objectives included the optimization of culture conditions for biomass production, the characterization of cultivar- and medium-dependent metabolite accumulation patterns, and the identification of compounds potentially associated with skin-protecting properties, including flavan-3-ols, phenolic acids, and stilbenoids. Attention was paid to the production of antioxidant and depigmenting constituents that may contribute to the prevention of oxidative stress-related skin damage and hyperpigmentation. To achieve this goal, comprehensive UPLC–MS analysis was combined with antioxidant (DPPH, ABTS, FRAP) and anti-tyrosinase assays. The selected analyses were used to provide a complete picture of both the chemical composition and biological properties of extracts. The determination of phenolic compounds along with antioxidant and anti-tyrosinase assays allowed the examination of whether the accumulation of specific metabolites is, related to activities that may be relevant for potential cosmetic application. Comparisons of six grapevine cultivars under different culture conditions were performed to characterize differences in their metabolite profiles and biological activities. This approach provided a broader valuation of their potential as biotechnology-derived ingredients for skin-care formulations.

The study aimed not only to determine the phytochemical and biological value of grapevine callus extracts but also to identify the most promising cultivars for future applications. By linking metabolite production with cosmetologically relevant bioactivity, this work provides new insight into the use of *V. vinifera* callus cultures as a sustainable, standardized, and innovative source of active ingredients for advanced skin-care formulations.

## 2. Results

The established callus cultures exhibited distinct morphological characteristics and growth responses depending on both grapevine cultivar and culture medium compositions. Furthermore, considerable differences were observed in the accumulation of bioactive metabolites and in the biological activities of the obtained extracts.

### 2.1. Morphology and Biomass Production of Experimental Callus Cultures

**Figure 1:**
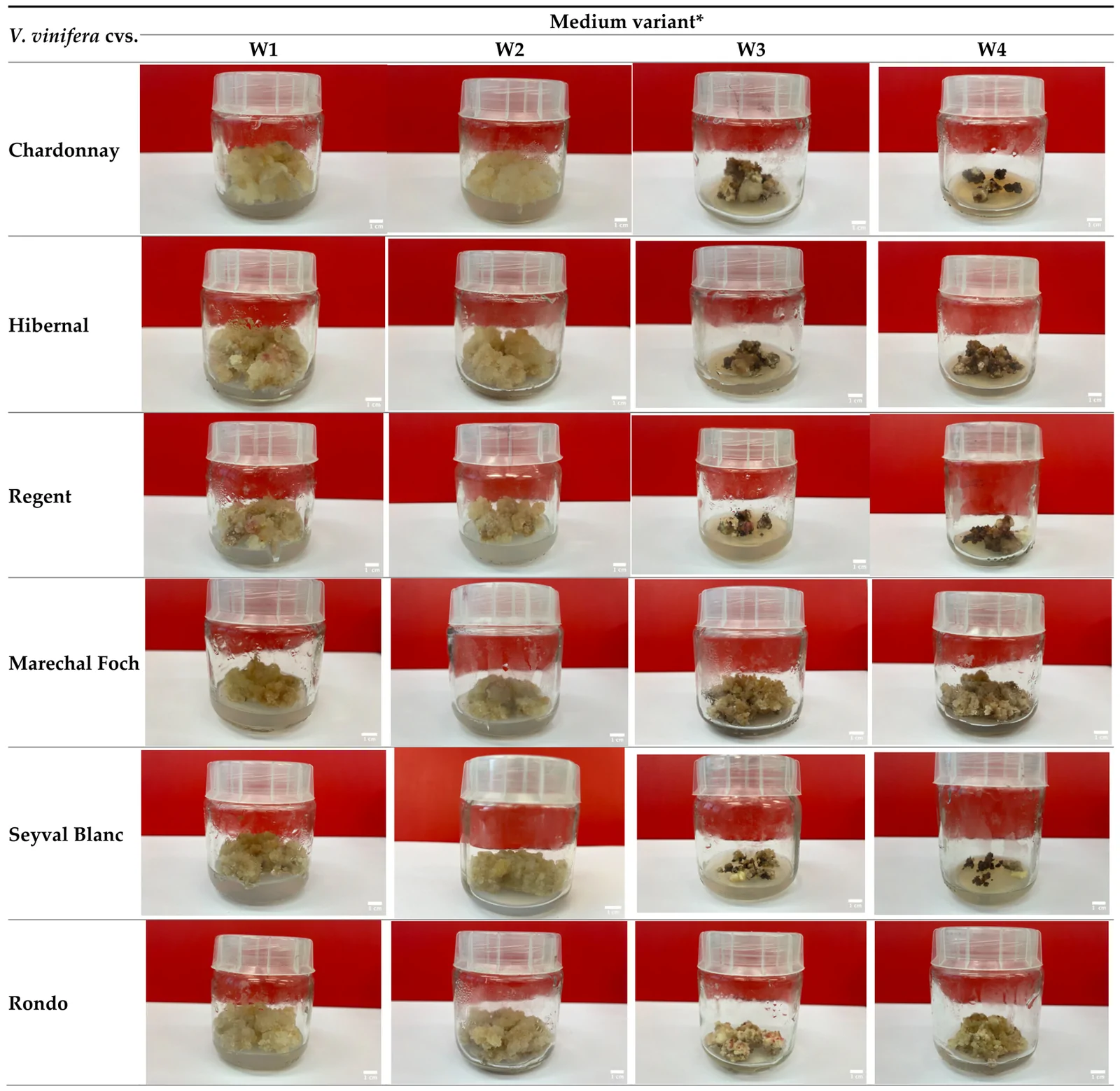
Visual comparison of callus cultures of *V. vinifera* cvs. Chardonnay, Hibernal, Regent, Marechal Foch, Seyval Blanc, and Rondo after 30 days of growth under different culture conditions. The photographs illustrate cultivar-dependent differences in biomass development and callus morphology as influenced by the composition of basal medium and plant growth regulators (* ‘W1’—MS, 0.9 mg/L BA and 0.1 mg/L IBA, ‘W2’—MS, 1.5 mg/L BA and 0.2 mg/L NAA, ‘W3’—SH, 0.9 mg/L BA and 0.1 mg/L IBA, ‘W4’—SH, 1.5 mg/L BA and 0.2 mg/L NAA).

Visual assessment of the cultures revealed noticeable differences in callus morphology depending on both cultivar and culture medium. Calli grown on MS medium (W1 and W2) generally appeared more compact and vigorously developed, whereas cultures maintained on SH medium (W3 and W4) tended to show a looser structure and lower biomass development. Differences in color intensity, texture, and callus organization were also observed among cultivars, indicating genotype-dependent responses to the applied culture conditions. Overall, the morphological observations corresponded well with the growth index values recorded for the individual culture variants (Figure 1).

Callus cultures of all six cultivars exhibited intensive biomass proliferation during the 30-day culture cycle. The highest Gi values were recorded on MS medium (W1 and W2), reaching 96.82 for cv. Chardonnay and 96.27 for cv. Hibernal. In comparison, cultures grown on SH medium (W3 and W4) generally produced lower biomass, particularly in cv. Regent (87.91 and 88.09) and cv. Seyval Blanc (88.22 and 89.49). The growth index was significantly affected by both cultivar (*p* < 0.05) and culture medium (*p* < 0.05), with response to the tested media varying among cultivars. Overall, the MS-based medium (W1 and W2) supported higher Gi values than SH-based medium (W3 and W4) in most cultivars, whereas no significant differences among the tested media were observed for cv. Marechal Foch (Table 1).

### 2.2. Phytochemical Profiling of Callus Extracts

The phytochemical profile of the extracts was characterized by UPLC–MS, leading to the identification of 12 compounds, including two amino acids (i.e., *L*-isoleucine, *L*-tryptophan), one phenolic acid (i.e., gallic acid), three flavan-3-ols (i.e., catechin, epicatechin, procyanidin B3, procyanidin B4), three monomeric stilbenoids (i.e., piceatannol, piceid, *E*-resveratrol), and two dimeric stilbenoids (i.e., ampelopsin, *E*-*ε*-viniferin).

Among the identified flavan-3-ols, procyanidins B3 and B4 represent B-type dimeric proanthocyanidins composed of flavan-3-ol units connected through a C4 → C8 interflavan bond. Procyanidin B3 is composed of two (+)-catechin units [catechin-(4α → 8)-catechin], whereas procyanidin B4 consists of (+)-catechin and (−)-epicatechin [catechin-(4α → 8)-epicatechin]. Thus, the two compounds differ in the stereochemistry of the terminal flavan-3-ol unit [38].

The identified stilbenoids comprised both monomeric and dimeric forms. Stilbenoids are a group of phenolic compounds naturally occurring in grapevine and involved, among others, in plant defense responses. Monomeric stilbenoids consist of a single stilbene unit and include compounds such as *E*-resveratrol and its derivatives, whereas dimeric stilbenoids are formed by oxidative coupling of two stilbene units and include compounds such as viniferins. Such oligomerization increases structural diversity and may also influence their biological properties [39].

Metabolite analysis was performed using a targeted UPLC-MS/MS approach, screening a broad range of compounds, as described in previously published articles [37,40,41,42]. The present grapevine callus cultures exhibited a more restricted profile of secondary metabolites than previously observed in differentiated grapevine tissues, including shoot cultures, canes, leaves and berries.

UPLC-MS analysis of the grapevine extracts revealed considerable differences in the accumulation of the analyzed metabolites depending on both the cultivar and the composition of the culture medium. The complete dataset for the individual metabolites is provided in Table 2, while the total concentrations of the main metabolite classes are presented in Table S1.

Results are expressed as mean ± SD. Different superscript letters within the same cultivar indicate statistically significant differences between variants (*p* < 0.05), with the letter ‘a’ corresponding to the highest mean value.

Stilbenoids represented one of the most abundant groups of compounds detected in the analyzed extracts. Among the identified compounds, only monomeric and dimeric stilbenoids were quantified. Their accumulation varied markedly among the tested combinations of cultivars and culture conditions. Depending on the cultivar, their total concentration ranged from 0.46 mg/g DW to 424 mg/g DW (Table S1). Particularly interesting differences were observed for individual stilbenoid compounds. For instance, *E*-*ε*-viniferin, a characteristic stilbenoid associated with *Vitis vinifera* L., reached its highest concentration in Seyval Blanc cultivated in W2 medium. A pronounced accumulation of *E*-resveratrol was also observed in Marechal Foch (180 mg/g DW), cultured in W1 medium. However, the total stilbenoid content in W3 and W4 media appeared to provide more favorable conditions for overall stilbenoid accumulation.

Flavan-3-ols constituted another important group of phenolic compounds detected in the extracts. Their total concentration varied considerably among the investigated cultivars and culture conditions. The highest value was recorded for cv. Seyval Blanc cultivated in W4 medium, reaching 136.52 mg/g DW. Catechin was among the most abundant flavan-3-ols detected in the analyzed extracts. A particularly high concentration of epicatechin was found in cv. Seyval Blanc cultivated in W2 medium, reaching 81.30 mg/g DW (Table 2). Interestingly, cv. Chardonnay (W4) and cv. Marechal Foch (W3) were the only cultivars, which promoted the significant accumulation of procyanidin B4 reaching 44.28 and 30.50 mg/g DW, respectively. The pronounced differences observed between cultivars, even under comparable culture conditions, suggest that the accumulation of individual flavan-3-ols was strongly associated with the analyzed grapevine genotypes.

Moreover, gallic acid was detected and quantified in the analyzed callus extracts, with the highest concentration observed in cv. Marechal Foch. The concentration reached 338 and 345 mg/g DW in calluses cultivated in W1 and W2 media, respectively. Such a pronounced accumulation contributed substitutionally to the overall phenolic profile of this cultivar and distinguished Marechal Foch from other investigated genotypes.

In addition to phenolic compounds, the analyzed extracts contained amino acids, although their concentrations were considerably lower than those observed for some groups of phenolic metabolites. The total amino acids ranged from 0.45 to 10.87 mg/g DW, depending on the cultivar and culture medium. The observed distribution suggests that W1 and W2 conditions generally favored amino acid accumulation, whereas W3 and W4 appeared to be less favorable for its biosynthesis.

The total concentration of quantified metabolites varied substantially among the investigated cultivars. The lowest total metabolite accumulation was generally associated with cv. Rondo (1.33–82.81 mg/g DW). In contrast, cv. Marechal Foch exhibited the highest overall accumulation of the analyzed metabolites, reaching 756.85 mg/g DW in W1 medium (Table S1).

### 2.3. Biological Activities of Callus Extracts

#### 2.3.1. Antioxidant Activity Assays

To determine the antioxidant activity of the obtained extracts the DPPH, ABTS and FRAP assays were used. Values are expressed as Trolox Equivalent Antioxidant Capacity (TEAC). The results of the DPPH assay showed that all tested extracts exhibited antioxidant activity related to various scavenging mechanisms (Table 3). Among all tested extracts, the best results were obtained for cv. Marechal Foch and cv. Seyval Blanc with values of 0.88 and 0.86 mM TE/L, respectively. The lowest DPPH scavenging activity was observed for cv. Regent with values ranging from 0.12 to 0.27 mM TE/L depending on the culture medium. The results of ABTS assay showed that all tested extracts have a high ability to scavenge the free radicals and their activities were all comparable independently from cultivation medium (Table 3). However, in the case of FRAP assay, the ferric-reducing activity was culture- and medium-dependent (Table 3). The highest results were obtained for cv. Seyval Blanc cultured in W1 and W2 media with values of 3.10 and 2.88 mM TE/L, respectively. The lowest ferric-reducing capacity was observed for cv. Regent cultured in W1 medium.

#### 2.3.2. Enzyme Inhibition Activity Assay

In tyrosinase inhibition assay, all tested extracts were potent and showed significant inhibition activity (Table 4). No significant correlation was found between anti-tyrosinase inhibition and type of medium. Similarly, the grapevine cultivar did not significantly affect the extracts’ ability to inhibit tyrosinase, although the highest anti-tyrosinase potential was observed for cv. Chardonnay cultured in W1 medium, which demonstrated the greatest enzyme-inhibiting activity with a value of 65.06%. Notably, cv. Rondo cultured in W3 medium exhibited the lowest skin-whitening potential (46.60%).

### 2.4. Principal Component Analysis (PCA)

**Figure 2:**
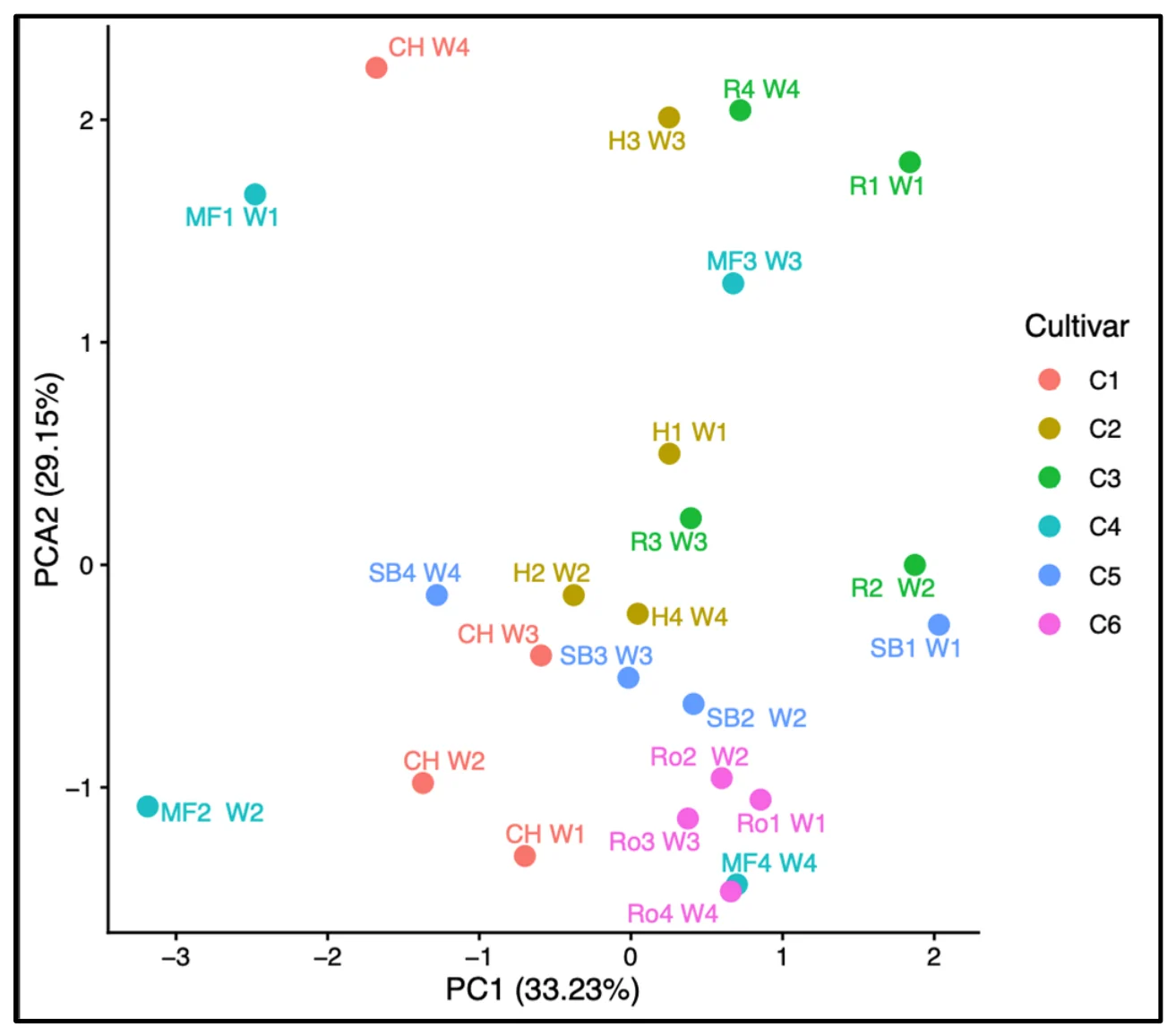
Principal component analysis (PCA) of *V. vinifera* cvs. Chardonnay, Hibernal, Regent, Marechal Foch, Seyval Blanc, and Rondo after 30 days of growth under different culture conditions (‘W1’—MS, 0.9 mg/L BA and 0.1 mg/L IBA, ‘W2’—MS, 1.5 mg/L BA and 0.2 mg/L NAA, ‘W3’—SH, 0.9 mg/L BA and 0.1 mg/L IBA, ‘W4’—SH, 1.5 mg/L BA and 0.2 mg/L NAA). C1—Chardonnay; C2—Hibernal; C3—Regent; C4—Marechal Foch; C5—Seyval Blanc; C6—Rondo.

The principal component analysis (PCA) was performed to identify the main differences in metabolite profiles of *V. vinifera* callus culture extracts obtained from different cultivars and cultivated under varying culture conditions. According to the PCA plot (Figure 2), most of the cultivars showed a relatively dispersed pattern, which indicates differences in the overall metabolite profiles among samples and suggests variability in their phytochemical composition. In contrast, the samples of cv. Rondo were located relatively close to each other, indicating a more consistent and similar metabolite profile across the tested culture media.

**Figure 3:**
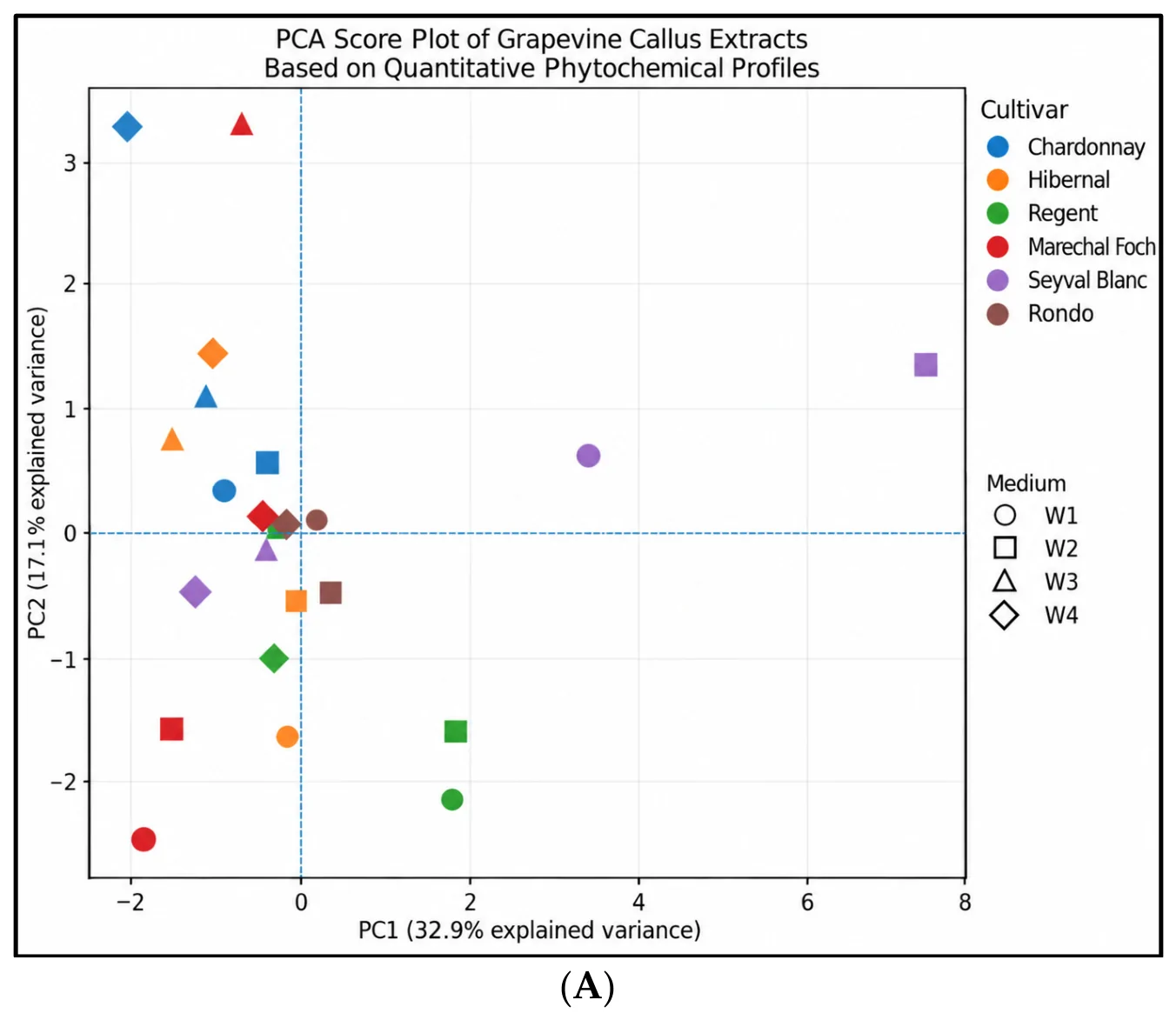

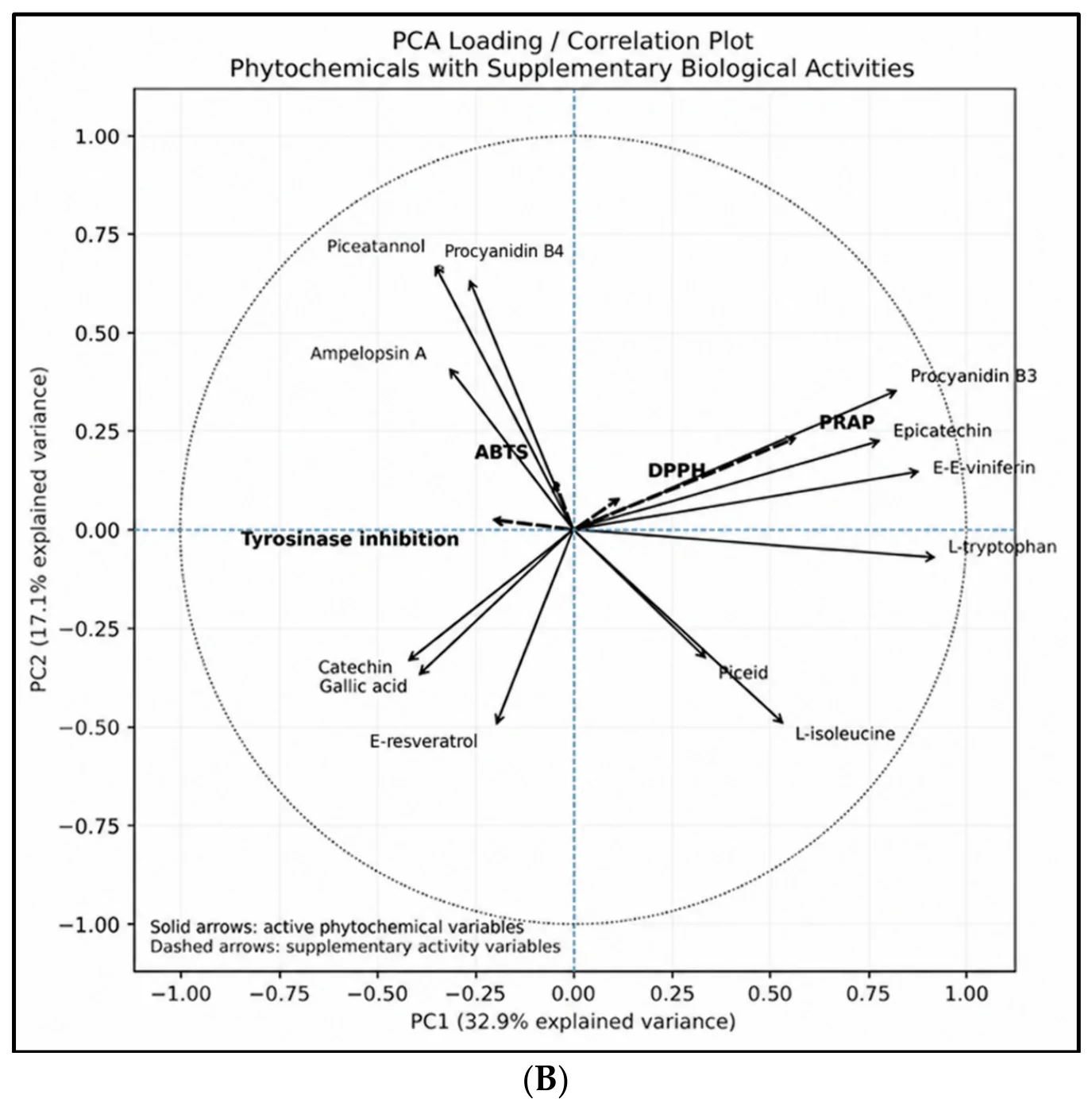
Principal component analysis (PCA) of quantitative phytochemical profiles of grapevine callus extracts cultured on four medium variants (W1–W4): (**A**) PCA score plot showing the distribution of 24 cultivar–medium combinations; (**B**) PCA loading/correlation plot for the 12 quantified phytochemicals (‘W1’—MS, 0.9 mg/L BA and 0.1 mg/L IBA, ‘W2’—MS, 1.5 mg/L BA and 0.2 mg/L NAA, ‘W3’—SH, 0.9 mg/L BA and 0.1 mg/L IBA, ‘W4’—SH, 1.5 mg/L BA and 0.2 mg/L NAA).

UPLC-MS data were averaged across three replicates for each combination, mean-centered, and unit-variance-scaled prior to PCA. PC1 and PC2 explained 32.9% and 17.1% of the total variance, respectively, accounting for 50.0% (Figure 3) of the cumulative variance. Colors indicate grapevine cultivars and symbols indicate medium variants. ABTS, DPPH, FRAP and tyrosinase inhibition were projected as supplementary variables and therefore did not contribute to the construction of the principal components. Variables oriented in similar directions indicate positive associations, whereas opposite directions indicate negative associations. Variables located close to the origin are weakly represented by the PC1–PC2 plane.

PCA revealed clear differentiation in the phytochemical profiles of grapevine callus extracts depending on cultivar and medium combinations. Procyanidin B3, epicatechin, *E*-*ε*-viniferin, *L*-tryptophan, catechin, gallic acid, and *E*-resveratrol contributed substantially to sample differentiation. The antioxidant activities measured by ABTS, DPPH and FRAP, as well as tyrosinase inhibition, were associated with specific phytochemical profiles, indicating that variations in biological activity were related to differences in secondary metabolite composition. Overall, both cultivar and culture medium influenced the phytochemical profile and biological activity of the extracts, with the effect of the medium being cultivar-dependent. Interestingly, antioxidant capacity measured by FRAP was associated with higher levels of procyanidin B3, epicatechin, *E*-*ε*-viniferin and *L*-tryptophan in the cultivar Seyval Blanc under the culture conditions W1 (MS, 0.9 mg/L BA and 0.1 mg/L IBA) and W2 (MS, 1.5 mg/L BA and 0.2 mg/L NAA).

## 3. Discussion

Skin aging is a natural and complex process influenced by various internal and external factors. It involves gradual changes in skin structure and function, leading to visible signs such as loss of elasticity, wrinkles, and pigmentation [1,43,44,45]. Therefore, the search for natural compounds that may help maintain skin condition and protect it from damage is of growing interest in cosmetic research. Phytochemicals are recognized for their diverse and often valuable pharmacological and biological properties. Nevertheless, their accumulation in plant tissues is not constant and may be considerably modified by environmental conditions leading to differences in the chemical composition and bioactivity of the resulting material. Plant biotechnology offers an opportunity to regulate these factors and consequently to obtain biomass with a desirable metabolite profile [46,47,48,49]. In this study, two commonly applied culture media, Murashige and Skoog (MS) and Schenk and Hildebrandt (SH), in combination with various plant growth regulators (PGRs), were investigated for its influence on chemical composition and biological activity of extracts obtained from the cultured biomass.

Among the tested extracts, Murashige and Skoog medium provided the most favorable environment for biomass accumulation and prolonged maintenance of callus cultures. The comparable growth index values recorded for W1 and W2 suggest that different combinations of plant growth regulators had only a limited effect on callus proliferation under these conditions. In contrast, cultures maintained on MS medium showed higher Gi values than those grown on Schenk and Hildenbrandt medium (W3 and W4). This is consistent with previous research, where W2 medium appeared to be the most favorable for shoot development. Conversely, the consistently higher Gi values observed on MS medium demonstrate that the basal nutrient composition was the main factor affecting callus growth and biomass accumulation in the studied cultivars (Table 1). Moreover, the highest results were recorded both for cv. Chardonny callus and for the shoots with values of 96.82 and 95.89, respectively [37]. The superior performance of MS medium may be attributed to its higher total salt concentration, particularly its increased nitrogen, potassium, and micronutrient content compared with SH medium, which could promote more intensive cell proliferation and biomass accumulation. However, further studies employing modified media formulations would be necessary to identify which individual medium components are principally responsible for these effects.

Previous studies have demonstrated that callus derived from different explant sources may differ in their accumulation of bioactive compounds [50,51,52,53]. Therefore, the endogenous metabolic background of the explant should be considered as a potential factor contributing to differences in callus formation and biochemical composition. In the present study only stem segments were used as explants. Consequently, the potential effect of explant source could be evaluated. Further comparative studies using different explant types would enrich the present findings and provide a more comprehensive understanding of the factors influencing callus induction growth and metabolite production.

Moreover, it should be noted that the PGR screening performed in the present study was limited to the selected combinations and concentrations of the two tested plants growth regulators; as no systemic gradient concentration screening was conducted, the tested PGR rations should not be considered as optimal conditions for metabolite production in the callus cultures. The present results demonstrated the effect of the selected PGR combinations, but the possibility that other concentrations of PGR may result in a higher accumulation of the target metabolites could not be excluded. Therefore, more comprehensive optimization approach involving a broader range of PGR concentrations and combinations is required to identify the optimal conditions for metabolite production.

The application of UPLC-MS analysis enabled the identification of 12 compounds from different chemical groups, e.g., amino acids, phenolic acids, flavan-3-ols, and stilbenoids (Table 2 and Table S1). The concentration of identified compounds was dependent on culture medium, which is consistent with previous research [37]. In the case of shoots, amino acids constituted the predominant group of the identified compounds, with total concentrations ranging from 12.39 to 90.42 mg/g DW [37], while in callus cultures their total concentrations ranged from 0.45 to 10.87 mg/g DW, depending on the cultivar. As the present study did not specifically assess the contribution of individual amino acids to the observed biological activities, these metabolites were not further considered in the context of the antioxidant and skin-protective properties of the extracts.

Therefore, stilbenoids were the major constituents detected in callus cultures. The accumulation of stilbenoids in callus cultures may be particularly relevant due to the diverse biological activities attributed to this group of compounds, including antioxidant, anti-inflammatory, or skin-whitening properties [39]. Notably, certain cultivars maintained under specific culture conditions showed a marked increase in the accumulation of valuable stilbenoids. For instance, cv. Marechal Foch cultured in W1 medium showed a pronounced accumulation of *E*-resveratrol, one of the most extensively studied grapevine stilbenoids (180 mg/g DW). In the case of shoots, its concentration reached 0.16 mg/g DW [37]. Moreover, the accumulation of *E*-resveratrol in callus cultures is significantly higher compared to that observed in the parent plant. For instance, it was reported that the concentrations of *E*-resveratrol in grape pomaces of four different cultivars such as Negroamaro, Malvasia di Lecce, Primitivo, and Cabernet Sauvignon were 0.10, 0.08, 0.12 and 0.09 mg/g DW, respectively [54]. In another study, the accumulation of *E*-resveratrol in grape pomace achieved the values of 0.0050–0.0069 mg/g DW [55]. The concentrations of *E*-resveratrol in Chardonnay pomace and seeds were 0.0263 and 0.0063 mg/g DW, respectively [56]. Besides *E*-resveratrol, callus cultures appeared to be a rich source of *E*-*ε*-viniferin, a resveratrol dimer, known for its diverse biological activities. The particularly high concentrations were exhibited by cv. Seyval Blanc cultivated in W1 and W2 media (54.63 and 56.93 mg/g DW, respectively). These findings suggest that both cultivar genotype and culture conditions may strongly influence the biosynthesis and accumulation of specific bioactive compounds. Compared to shoots [37], callus cultures also exhibited higher concentrations of flavan-3-ols, which are of particular interest due to their strong antioxidant properties in neutralizing reactive oxygen species [57,58]. For instance, the concentration of catechin in shoots reached 2.73 mg/g DW [37], whereas in the case of callus the highest concentration was achieved by Seyval Blanc (W4) (136.51 mg/g DW). Moreover, compared to the Chardonnay pomace and seeds the concentrations of catechin were 0.67 and 3.39 mg/g DW, respectively [56]. In another study, concentrations of catechin in grape pomace reached 9.9–16.1 mg/g DW [55,59]. Interestingly, cv. Seyval Blanc callus cultured in W2 medium showed the highest accumulation of epicatechin among all tested extracts. For comparison, the concentration of epicatechin in Chardonnay reached 0.89 mg/g DW in pomace and 9.4 mg/g DW in seeds [56].

Gallic acid concentration in grape pomace reached 252.8 µg/g DW [60], whereas in the case of callus cultures its concentration was 345.69 mg/g DW.

Consequently, the highest content in the above-mentioned group of compounds observed in callus biomass suggests that it may represent a valuable source of bioactive compounds with potential applications in skin-care formulations.

The previous studies on *Vitis vinifera* L. have mainly focused on shoots, whereas the present study demonstrates how variations in callus culture conditions may influence metabolite accumulation and the resulting biological activity. The previous observations showed that shoots generally contained lower metabolite levels and exhibited lower biological activity than corresponding callus cultures. In contrast, callus cultures accumulated higher amounts of the investigated metabolites, accompanied by increased biological activity. These findings suggest that callus cultures may serve as an effective system for enhancing secondary metabolite production in grapevines.

To determine antioxidant activity, the DPPH, ABTS and FRAP assays were performed. The results indicate that all tested extracts exhibit radical-scavenging activity dependent on the applied media. Notably, the antioxidant activity of tested extracts could be attributed to its rich phytochemical composition. The result of DPPH assay showed that cvs. Seyval Blanc and Marechal Foch cultivated in W3 medium possessed the highest activity (0.86 mM TE/L and 0.88 mM TE/L, respectively). This finding may be attributed to enhanced accumulation of stilbenoids identified in the cultivars (Table 2), particularly ampelopsin, piceatannol and piceid. The results of ABTS assay showed that all extracts exhibited comparable activity (Table 4).

The differences observed among the three antioxidant assays reflect the different chemical principles underlying each assay. The DPPH and ABTS assays showed relatively similar trends among the investigated samples, whereas the FRAP results differed from those obtained using the radical-scavenging assays. This discrepancy can be explained by the different reaction mechanisms underlying these analytical models. Both DPPH and ABTS assays primarily evaluate the ability of compounds to neutralize the stable DPPH and ABTS radicals through electron transfer reactions, whereas FRAP measures the reducing capacity of an extract based on the reduction of Fe^3+^ to Fe^2+^. Therefore, compounds present in the extracts may exhibit different reactivities toward the radical species used in DPPH and ABTS assays compared with the ferric ion reduction system employed in the FRAP assay. The observed differences may also be related to the qualitative and quantitative composition of phenolic metabolites in the extracts. Flavonoids, phenolic acids, stilbenoids, and other phenolic compounds can differ in their electron-donating and radical-scavenging capacities depending on their chemical structure, including the number and position of hydroxyl groups. Consequently, individual metabolites may exhibit different relative activities depending on the assay system. This aspect may be particularly relevant to the present extracts, which contained several structurally diverse metabolites. For instance, gallic acid is a particularly strong reducing phenolic compound and its high reducing capacity has been associated with the presence of three hydroxyl groups on the aromatic ring [61]. Therefore, differences in the relative abundance of gallic acid among the samples may contribute to differences in FRAP values. Since the extract contained a heterogeneous composition of metabolites, which differ in their chemical structure and antioxidant properties, their synergistic effect may additionally contribute to the overall biological activity.

The tyrosinase inhibition assay was performed to investigate the skin-whitening activity of the tested cultivars. The best results in tyrosinase inhibition were obtained for cv. Chardonnay cultivated in W1 medium (above 60% inhibition). However, in the case of shoots the percentage inhibition reached 17% [38]. Such differences could be attributed to higher concentrations of both flavan-3-ols and stilbenoids, which are proven to possess anti-tyrosinase activity [62,63].

It is important to mention that the antioxidant and tyrosinase inhibition activities were evaluated using an in vitro approach, which provides useful information regarding the antioxidant capacity of the tested extracts and potential ability to inhibit tyrosinase under defined experimental conditions. However, further studies using relevant skin models are required to determine the cellular relevance of these activities.

The PCA showed that several phenolic compounds contributed to the separation of the extracts for PC1. Procyanidin B3, epicatechin and *E*-*e*-viniferin were associated with the positive PC1 direction, whereas catechin, gallic acid and *E*-resveratrol were associated with the negative direction, together with tyrosinase inhibition activity. Along PC2, piceatannol, ampelopsin A and procyanidin B4 were associated with the region of the ABTS variable, suggesting a possible contribution to the antioxidant activity. The DPPH and FRAP results were also associated with specific combinations of phytochemicals rather than with a single compound. Overall, the PCA indicates that differences in phytochemical composition were associated with differences in biological activity. However, these associations do not demonstrate direct relationships as the observed activities may result from the combined effects of several metabolites.

Considering the large number of *V. vinifera* cultivars, many of which have not been investigated, there is a potential to identify and select the most promising cultivar based on their chemical composition and biological activity, potentially enabling the development of more standardized cosmetic ingredients.

## 4. Materials and Methods

### 4.1. Chemicals and Reagents 

The reagents used for the maintaining of in vitro cultures, including Murashige and Skoog (MS) and Schenk and Hildebrandt (SH) media, sucrose, agar, and plant growth regulators (PGRs), were supplied by Duchefa Farma B.V. (Haarlem, The Netherlands). Ultrapure water was obtained using a Milli-Q water system (Merck Millipore, Molsheim, France). LC-MS-grade acetonitrile and formic acid, as well as analytical-grade methanol, were obtained from Fisher Scientific (Illkirch-Graffenstaden, France). Reference standards of *L*-isoleucine, *L*-tryptophan, gallic acid, catechin, epicatechin, *E*-resveratrol, *E*-piceid and *E*-piceatannol and ampelopsin A were purchased from Sigma-Aldrich (St. Louis, MO, USA). Procyanidin B3 and procyanidin B4 were obtained from Extrasynthese (Genay, France), and *E*-*ε*-viniferin was purchased from BLDpharm (Reinbek, Germany).

The reagents used for biological activities assays, including DPPH, ABTS, TPTZ, Trolox, tyrosinase, phosphate buffer, *L*-DOPA, and kojic acid, were purchased from Sigma-Aldrich.

### 4.2. Initiation of Experimental Callus Cultures

Briefly, young stem explants of *V. vinifera* cvs. Chardonnay, Hibernal, Regent, Marechal Foch, Seyval Blanc, and Rondo, harvested in August 2022 in ‘Nad dworskim potokiem’ vineyard (Łazy 2, 32-765 Rzezawa, Poland, 49°58′00.8″ N, 20°29′54.1″ E), were subjected to sterilization using a 1% chloramine for 8 min, followed by triple rinsing in sterile redistilled water to eliminate disinfectant residues. The grapevine cultivars included in this study were selected to represent genetically and phenotypically diverse cultivars differing in their origin and characteristics, thereby allowing the influence of genotype on the production of phenolic metabolites under in vitro conditions to be evaluated.

The size of stem segments was 1–1.5 cm. The explants were cultivated in vessels containing 30 mL of culture medium, with one explant placed in each culture vessel. During the initiation stage, the explants were cultured on Murashige and Skoog [64] medium without plant growth regulators.

Cultures were cultivated under controlled growth conditions (25 ± 2 °C; relative humidity approx. 60–70%), under continuous illumination provided by white LED lamps, with a photosynthetic photon flux density (PPFD) of approximately 45 µmol m^−2^ s^−1^. For biomass production, previously established callus cultures, with an initial inoculum of 2.0–2.5 g of fresh weight, were transferred to vessels containing 30 mL of culture medium and subcultured in 30-day growth cycles in different variants, Murashige and Skoog and Shenk and Hildebrandt media [65], supplemented with different types and concentrations of plant growth regulators (PGRs). The medium variants were designated as ‘W1’ for MS, 0.9 mg/L 6-benzylaminopurine (BA) and 0.1 mg/L indole-3-butyric acid (IBA), ‘W2’ for MS, 1.5 mg/L BA and 0.2 mg/L 1-naphthylacetic acid (NAA), ‘W3’ for SH, 0.9 mg/L BA and 0.1 mg/L IBA, and ‘W4’ for SH, 1.5 mg/L BA and 0.2 mg/L NAA. For each cultivar and medium variant, four experimental series were established, with three culture vessels per series. Following the second 30-day subculture period, the obtained biomass was collected, frozen, and freeze-dried using a lyophilizer (Martin Christ Gefriertrocknungsanlagen GmbH, Osterode am Harz, Germany). The increase in biomass was determined based on dry weight measurements using the growth index (Gi), which was calculated according to the following equation [66]:

$$\mathrm{Gi}=\frac{\left(Dw_{n}-Dw_{0}\right)}{Dw_{n}}\times 100$$

where Gi represents the growth index, Dw_0_ refers to the dry weight of the inoculum and Dw_0_ corresponds to the dry weight measurements after (n) days.

### 4.3. Preparation of Callus Extracts

The lyophilized biomass derived from *V. vinifera* cv. callus cultures were subjected to ultrasound-assisted (Hettig, Solingen, Germany) extraction using 70% ethanol as a solvent. The extraction was carried out at a plant material-to-solvent ratio of 1 g/10 mL. Sonication was performed for 30 min at 25 °C. Following extraction, the mixture was filtered and the solvent evaporated. The remaining extract was freeze-dried to obtain a dry extract, which was subsequently used for further studies. All obtained extracts were prepared according to the procedure described.

### 4.4. Phytochemical Analysis of Callus Extracts

The UPLC–MS analysis was carried out using an ACQUITY™ Ultra Performance Liquid Chromatography system (Waters, Milford, MA, USA) coupled with a photo diode array detector (PDA) and a Xevo TQD mass spectrometer equipped with an electrospray ionization (ESI) source. UPLC-MS quantification method was based on previously developed method [37]. Instrumental control was performed by Masslynx 4.1 software (Waters, Milford, MA, USA). Chromatographic separation was achieved on a Waters Acquit HSS T3 C18 column (150 × 2.1 mm, 1.8 μm) maintained at 55 °, with a flow rate of 0.4 mL min^−1^ and injection volume of 5 μL. The mobile phase consisted of 0.1% formic acid in water (solvent A) and 0.1% formic acid in acetonitrile (solvent B). Separation was performed using linear gradient from 5% to 50% of solvent B over 18 min., followed by an 8 min column washing and reconditioning. Mass spectrometric analysis was carried out in both positive and negative ionization modes. The capillary voltage was set to 2 kV, while the sample cone voltages were set to 30 and 120 V. Cone and desolvation gas flow rates were adjusted to 30 and 1000 Lh^−1^, respectively. Metabolites were identified based on their retention times, mass spectra and UV spectra, using reference standards for comparison. Quantitative analysis was performed in selected ion monitoring (SIM) mode. To eliminate potential analytical variation, the injection sequence was randomized independent of genotype or culture conditions. No internal standard was used. The calibration standards and samples were analyzed in three independent replicates. For the analyses 2 mg of dry extract was placed in Eppendorf and mixed with 1 mL of 70% ethanol (*v*/*v*). Metabolite concentrations were calculated individually using the corresponding external calibration curves and mean peak areas from replicate injections. The calculated concentrations were corrected for dilution, extraction volume, and sample dry mass, and expressed as mg/g DW. The total metabolite content was calculated as the sum of the concentrations of all individual metabolites identified within the respective metabolite groups.

### 4.5. Biological Evaluation of Callus Extracts

As part of the biological evaluation of the obtained callus extracts, antioxidant activity was determined using DPPH, ABTS, and FRAP assays, and skin-whitening properties were assessed by measuring tyrosinase inhibition.

#### 4.5.1. Study of Antioxidant Activity of Callus Extracts

The free radical-scavenging activity was evaluated using the DPPH method according to the procedure described in previous research [37]. For the analysis, 10 μL of each extract was pipetted into a 96-well plate and mixed with 190 μL of freshly prepared 0.1 mM DPPH reagent. After incubation (15 min at 25 °C), the absorbance was measured at 517 nm using a microplate reader (Infinite 200 PRO, Tecan, Switzerland). Trolox (6-hydroxy-2,5,7,8-tetramethylchroman-2-carboxylic acid) at concentrations of 0.05–0.8 mM/L was used to prepare the calibration curve, while the reference samples consisted of ethanol mixed with DPPH solution without extracts. The antioxidant capacity was determined from three independent experiments and expressed as Trolox Equivalent Antioxidant Capacity (TEAC).

Additionally, the antioxidant activity was also performed using the ABTS assay following the protocol reported by [67] with slight modifications. For this purpose, the ABTS reagent was prepared by dissolving ABTS in deionized water to obtain a concentration of 7 mM. The radical cation ABTS+ was generated by adding potassium persulphate to a final concentration of 2.45 mM. The resulting mixture was kept at 25 °C in the dark for 12 h to allow complete radical-scavenging formation. Prior to analysis, the ABTS+ solution was diluted in water until an absorbance of 0.7 was reached at 734 nm. Then, 10 μL of each extract was mixed with 190 μL of freshly prepared ABTS working solution in a 96-well plate and incubated for 15 min at 25 °C. The absorbance was measured at a wavelength of 734 nm using a microplate reader (Infinite 200 PRO, Tecan, Switzerland). Trolox (at concentrations of 0.05–0.8 mM/L) was used to prepare the calibration curve. Ethanol mixed with ABTS solution was used as a reference sample. The results were obtained from three independent experiments and expressed as TEAC.

The ferric-reducing capacity of extracts was determined according to [67] with slight modifications. The reagent consisted of TPTZ (10 mM), FeCl_3_·6H_2_O (20 mM), and acetate buffer (300 mM, pH 3.6) in a 1:1:10 (*v*/*v*/*v*) ratio. Each well of the 96-well plate contained 10 μL of each extract with 190 μL of freshly prepared FRAP solution and were incubated for 15 min at 25 °C. The absorbance was measured at a wavelength of 630 nm using a microplate reader (Infinite 200 PRO, Tecan, Switzerland). Trolox (0.05–0.8 mM/L) was used to prepare the calibration curve. The reference sample consisted of acetate buffer and FRAP solution without tested samples. The ferric-reducing power was calculated based on three independent experiments and expressed as mM Trolox Equivalent/L (TEAC).

#### 4.5.2. Tyrosinase Inhibition Activity

The tyrosinase inhibitory activity was determined according to the method described by [68,69]. Briefly, 50 µL of tested extract, 80 µL of phosphate buffer and 40 µL of tyrosinase working solution were transferred into a 96-well plate. After incubation (5 min at 25 °C), 40 µL of L-DOPA or phosphate buffer was added. The absorbance was measured at a wavelength of 475 nm using a microplate reader (Infinite 200 PRO, Switzerland, Tecan). The kojic acid (25–100 µg/mL) was used to prepare the calibration curve. The enzyme inhibition was calculated based on three independent experiments and expressed as percentage inhibition.

### 4.6. Statistical Analysis

Statistical differences among groups were assessed using two-way analysis of variance (ANOVA), followed by Tukey’s HSD post hoc test.

Principal component analysis (PCA) was used to visualize the overall variation in the phytochemical profiles of grapevine callus extracts and to assess relationships among samples and quantified compounds. In addition, correlation analysis was performed to evaluate associations between individual phytochemicals and biological activities.

The statistical analysis was performed using RStudio (version 2026.08.0+187) with R version 4.6.1 (24 June 2026).

## 5. Conclusions

This present study provides the first comprehensive comparative assessment of callus cultures derived from six *V. vinifera* cvs., namely Chardonnay, Hibernal, Regent, Marechal Foch, Seyval Blanc, and Rondo, with regard to their phytochemical composition and skin-care potential. The results demonstrate that grapevine callus cultures constitute an effective biotechnological platform to produce valuable phytochemicals and represent a promising alternative to conventionally sourced plant materials intended for dermatological applications.

The study demonstrated the significant cultivar- and medium-dependent differences in biomass proliferation, metabolite accumulation, and biological activity, confirming the importance of culture optimization for the targeted production of cosmetically relevant compounds.

UPLC–MS analysis revealed the presence of several classes of metabolites, particularly stilbenoids and flavan-3-ols, as well as phenolic acid (e.g., gallic acid), associated with antioxidant and photoprotective properties. The obtained phytochemical profiles indicate that grapevine callus cultures can produce compounds recognized as important functional ingredients in modern skin-care formulations.

The PCA demonstrated that both grapevine cultivar and culture medium significantly influence the phytochemical composition and biological activity of callus extracts.

Among the investigated cultivars, cv. Marechal Foch exhibited the highest biosynthetic capacity, accumulating total identified metabolites at a level of 756.85 mg/g DW. However, cv. Seyval Blanc was distinguished by a favorable balance between metabolite accumulation and biological activity. The strongest tyrosinase inhibitory activity was observed for Chardonnay extracts, highlighting their potential for use as skin-whitening and depigmenting bioactive ingredients.

Collectively, these findings identify cvs. Seyval Blanc, Chardonnay, and Marechal Foch as the most promising candidates for further cosmetic development. The demonstrated antioxidant and anti-tyrosinase activities confirm that grapevine callus-derived extracts retain biologically relevant properties that are directly applicable to skin-care and dermocosmetics products.

The study highlights the advantages of plant cell and tissue culture technologies to produce cosmetic ingredients with reproducible quality and controlled phytochemical composition. The results obtained demonstrate the potential of the in vitro cultivation system as a controlled platform for metabolite production, which may reduce the dependence of metabolite accumulation on seasonal environmental conditions. However, further validation using multiple independent explants samples collected at different time points and seasons is required to confirm the reproducibility of this potential advantage. Moreover, despite the valuable results obtained, further research involving scale-up studies, including cultivation in large-scale bioreactors, could represent an important step in the development of this approach toward potential industrial application.

In conclusion, this study provides evidence that plant callus cultures can serve as a valuable source of bioactive metabolites with potential relevance to skin-care applications. The metabolomic profile and biological properties of the investigated material indicate that callus-derived extracts may offer a promising basis for the development of functional cosmetic ingredients. Importantly, callus cultures represent a controlled and potentially sustainable alternative source of plant-derived bioactive compounds, allowing their production under standardized conditions. Future investigations should focus on validating the safety and efficacy of the most promising extracts in completed topical preparations and skin-cell models used to evaluate oxidative stress, melanogenesis, and inflammatory response. These studies may contribute to the future development of callus-derived ingredients for skin-care products.

## Figures and Tables

**Table 1:** Growth index (±SD) of callus cultures of various *V. vinifera* cvs. cultivated on different MS and SH medium variants (* ‘W1’—MS, 0.9 mg/L BA and 0.1 mg/L IBA, ‘W2’— MS, 1.5 mg/L BA and 0.2 mg/L NAA, ‘W3’—SH, 0.9 mg/L BA and 0.1 mg/L IBA, ‘W4’— SH, 1.5 mg/L BA and 0.2 mg/L NAA). Means within each cultivar (*n* = 3) followed by different letters differ significantly (one-way ANOVA followed by Tukey’s multiple comparison test (*p* < 0.05)).

*V. vinifera* cvs.	Medium Variant *			
	W1	W2	W3	W4
Chardonnay	95.48 ± 8.75 ^ab^	96.82 ± 8.93 ^a^	92.19 ± 2.51 ^b^	91.12 ± 1.13 ^b^
Hibernal	96.20 ± 9.02 ^a^	96.27 ± 6.85 ^a^	91.17 ± 0.92 ^b^	91.25 ±1.37 ^b^
Regent	95.75 ± 4.94 ^a^	95.45 ± 4.19 ^a^	87.91 ± 0.88 ^b^	88.09 ± 3.16 ^ab^
Marechal Foch	95.09 ± 4.31 ^a^	95.45 ± 5.01 ^a^	94.71 ± 4.39 ^a^	93.32 ± 3.05 ^a^
Seyval Blanc	95.02 ± 6.67 ^a^	95.85 ± 7.33 ^a^	88.22 ± 0.57 ^b^	89.49 ± 0.65 ^b^
Rondo	96.11 ± 7.48 ^a^	95.75 ± 6.39 ^a^	91.81 ± 1.86 ^b^	93.82 ± 2.87 ^c^

**Table 2:** The quantitative analysis of identified metabolites in the extracts of callus cultures of different *V. vinifera* cvs. Values are expressed as mg/g DW (±SD); nd *—not detected. ‘W1’—MS, 0.9 mg/L BA and 0.1 mg/L IBA, ‘W2’—MS, 1.5 mg/L BA and 0.2 mg/L NAA, ‘W3’—SH, 0.9 mg/L BA and 0.1 mg/L IBA, ‘W4’—SH, 1.5 mg/L BA and 0.2 mg/L NAA. Means within each cultivar (*n* = 3) followed by different letters differ significantly (one-way ANOVA followed by Tukey’s multiple comparison test (*p* < 0.05)).

*V. vinifera* cvs.	Medium Variant	*L*-Isoleucine	*L*-Tryptophan	Gallic Acid	Catechin	Epicatechin	Procyanidin B3	Procyanidin B4	Piceatannol	Piceid	*E*-Resveratrol	Ampelopsin A	*E*-*ε*-Viniferin
Chardonnay	W1	2.89 ± 0.93 ^a^	0.65 ± 0.17 ^a^	0.69 ± 0.19 ^a^	100.78 ± 9.00 ^a^	3.42 ± 0.52 ^a^	nd *	28.47 ± 4.88 ^a^	13.08 ± 0.50 ^a^	13.33 ± 0.97 ^a^	0.53 ± 0.09 ^a^	nd *	0.28 ± 0.04 ^a^
Chardonnay	W2	0.08 ± 0.02 ^b^	1.62 ± 0.18 ^b^	159.26 ± 82.08 ^b^	50.35 ± 10.63 ^b^	11.29 ± 0.37 ^a^	nd *	24.96 ± 7.53 ^a^	28.70 ± 0.25 ^a^	11.17 ± 0.63 ^b^	0.45 ± 0.04 ^a^	nd *	0.50 ± 0.04 ^ab^
Chardonnay	W3	0.39 ± 0.12 ^b^	0.12 ± 0.01 ^a^	71.06 ± 19.36 ^ab^	29.77 ± 10.30 ^b^	2.04 ± 0.55 ^a^	nd *	20.37 ± 1.50 ^a^	23.07 ± 8.62 ^a^	9.12 ± 0.01 ^c^	0.22 ± 0.01 ^b^	86.54 ± 27.88 ^a^	0.92 ± 0.36 ^b^
Chardonnay	W4	0.45 ± 0.06 ^b^	nd *	112.65 ± 44.02 ^ab^	51.62 ± 0.29 ^b^	7.14 ± 0.42 ^a^	nd *	44.28 ± 27.53 ^a^	101.31 ± 14.66 ^b^	8.21 ± 0.26 ^c^	0.51 ± 0.09 ^a^	310.02 ± 44.57 ^b^	4.92 ± 0.19 ^c^
Hibernal	W1	2.58 ± 1.06 ^a^	1.90 ± 0.30 ^a^	124.00 ± 40.31 ^a^	18.79 ± 7.11 ^a^	nd*	nd *	nd *	nd *	3.76 ± 0.39 ^a^	154.85 ± 20.73 ^a^	nd *	0.06 ± 0.02 ^a^
Hibernal	W2	nd *	1.19 ± 0.01 ^ab^	83.40 ± 28.62 ^a^	43.82 ± 6.93 ^b^	nd*	nd *	nd *	nd *	1.29 ± 0.14 ^b^	83.26 ± 29.11 ^a^	57.39 ± 4.88 ^a^	37.38 ± 2.61 ^b^
Hibernal	W3	0.81 ± 0.55 ^b^	0.43 ± 0.12 ^b^	41.55 ± 5.60 ^a^	18.63 ± 9.58 ^a^	nd*	nd *	nd *	78.58 ± 0.24 ^a^	0.43 ± 0.05 ^c^	121.47 ± 43.07 ^a^	187.10 ± 10.27 ^b^	1.66 ± 0.18 ^a^
Hibernal	W4	nd *	0.81 ± 0.16 ^b^	51.19 ± 7.08 ^a^	28.46 ± 7.96 ^ab^	nd*	nd *	nd *	106.78 ± 40.09 ^a^	0.99 ± 0.07 ^b^	0.48 ± 0.09 ^b^	nd *	2.61 ± 0.64 ^a^
Regent	W1	8.06 ± 2.96 ^a^	2.03 ± 0.32 ^a^	nd *	22.56 ± 0.28 ^a^	nd*	nd *	nd *	4.80 ± 0.17 ^a^	147.20 ± 2.80 ^a^	94.91 ± 38.80 ^a^	73.47 ± 16.89 ^a^	27.55 ± 2.04 ^a^
Regent	W2	6.08 ± 0.74 ^b^	4.22 ± 0.43 ^b^	nd *	13.54 ± 0.14 ^a^	6.73 ± 1.79 ^a^	nd *	nd *	nd *	141.80 ± 75.74 ^a^	0.11 ± 0.02 ^b^	nd *	0.04 ± 0.01 ^b^
Regent	W3	1.30 ± 0.08 ^c^	0.56 ± 0.02 ^c^	39.06 ± 5.56 ^a^	13.02 ± 0.28 ^a^	nd *	nd *	nd *	22.12 ± 5.93 ^b^	70.75 ± 25.71 ^a^	0.18 ± 0.06 ^b^	89.44 ± 8.84 ^ab^	1.91 ± 0.31 ^b^
Regent	W4	1.49 ± 0.04 ^c^	0.47 ± 0.13 ^c^	30.39 ± 0.12 ^a^	5.15 ± 0.97 ^a^	nd *	nd *	nd *	11.20 ± 2.07 ^a^	111.37 ± 33.92 ^a^	131.29 ± 8.35 ^a^	133.77 ± 28.70 ^b^	0.87 ± 0.06 ^b^
Marechal Foch	W1	2.56 ± 0.45 ^a^	0.63 ± 0.04 ^a^	338.97 ± 88.58 ^a^	101.69 ± 7.59 ^a^	nd *	nd *	nd *	5.76 ± 0.13 ^a^	8.59 ± 0.14 ^a^	180.03 ± 8.78 ^a^	118.19 ± 8.55 ^a^	0.43 ± 0.01 ^a^
Marechal Foch	W2	1.37 ± 0.40 ^a^	0.38 ± 0.07 ^a^	345.69 ± 58.12 ^a^	98.02 ± 33.81 ^a^	nd *	nd *	nd *	nd *	8.47 ± 1.19 ^a^	0.24 ± 0.04 ^b^	nd *	0.30 ± 0.02 ^a^
Marechal Foch	W3	1.75 ± 0.30 ^a^	0.24 ± 0.04 ^a^	0.79 ± 0.07 ^b^	0.66 ± 0.03 ^b^	0.19 ± 0.05 ^a^	0.22 ± 0.03 ^a^	30.50 ± 6.83 ^a^	49.11 ± 12.79 ^b^	30.66 ± 2.88 ^b^	1.31 ± 0.09 ^b^	227.89 ± 18.10 ^b^	3.72 ± 0.15 ^b^
Marechal Foch	W4	1.32 ± 0.23 ^a^	nd *	nd *	nd *	2.31 ± 2.12 ^a^	nd *	nd *	nd *	nd*	3.04 ± 2.45 ^b^	nd *	nd *
Seyval Blanc	W1	7.03 ± 0.36 ^a^	3.85 ± 0.59 ^a^	nd *	2.28 ± 0.46 ^a^	nd *	0.40 ± 0.04 a	nd *	11.61 ± 0.80 ^ab^	39.53 ± 0.79 ^a^	6.78 ± 0.70 ^a^	nd *	54.63 ± 9.30 ^a^
Seyval Blanc	W2	2.50 ± 0.19 ^b^	4.63 ± 0.11 ^b^	0.55 ± 0.15 ^a^	1.57 ± 0.06 ^a^	81.30 ± 29.70 ^a^	0.31 ± 0.05 a	nd *	0.13 ± 0.01 ^a^	28.47 ± 1.42 ^b^	6.19 ± 0.65 ^a^	20.23 ± 0.52 ^a^	56.93 ± 3.21 ^a^
Seyval Blanc	W3	0.36 ± 0.03 ^c^	0.91 ± 0.27 ^c^	99.79 ± 39.85 ^b^	0.64 ± 0.11 ^a^	nd *	nd *	nd *	7.72 ± 1.02 ^a^	3.09 ± 0.39 ^c^	0.25 ± 0.34 ^a^	85.04 ± 31.35 ^a^	1.00 ± 0.19 ^b^
Seyval Blanc	W4	1.27 ± 0.030 ^c^	0.34 ± 0.12 ^c^	44.92 ± 8.28 ^ab^	136.52 ± 18.59 ^b^	nd *	nd *	nd *	22.71 ± 7.67 ^b^	0.63 ± 0.14 ^c^	76.05 ± 0.13 ^b^	58.43 ± 1.92 ^b^	0.95 ± 0.17 ^b^
Rondo	W1	1.35 ± 0.09 ^a^	3.06 ± 0.98 ^a^	nd *	20.76 ± 3.83 ^a^	nd *	nd *	nd *	4.93 ± 0.17 ^a^	3.22 ± 0.27 ^a^	0.11 ± 0.24 ^a^	33.71 ± 1.54 ^a^	0.09 ± 0.03 ^a^
Rondo	W2	1.38 ± 0.18 ^a^	1.53 ± 0.38 ^b^	nd *	22.17 ± 2.09 ^a^	nd *	nd *	nd *	nd *	2.10 ± 0.13 ^a^	0.03 ± 0.12 ^a^	55.59 ± 3.12 ^b^	0.01 ± 0.01 ^a^
Rondo	W3	0.68 ± 0.07 ^a^	0.57 ± 0.35 ^c^	nd *	24.80 ± 2.21 ^a^	nd *	nd *	nd *	12.98 ± 0.51 ^b^	0.27 ± 0.11 ^a^	0.04 ± 0.02 ^a^	26.52 ± 0.12 ^a^	0.32 ± 0.01 ^a^
Rondo	W4	nd*	0.87 ± 0.01 ^bc^	nd *	nd *	nd *	nd *	nd *	0.12 ± 0.07 ^c^	0.13 ± 0.09 ^a^	0.03 ± 0.02 ^a^	nd *	0.17 ± 0.11 ^a^

**Table 3:** The free radical-scavenging activity extracts obtained from callus cultures of different *V. vinifera* cvs. using DPPH, ABTS, FRAP methods. Values are expressed as mM Trolox Equivalent/L (mM TE/L). * ‘W1’—MS, 0.9 mg/L BA and 0.1 mg/L IBA, ‘W2’—MS, 1.5 mg/L BA and 0.2 mg/L NAA, ‘W3’—SH, 0.9 mg/L BA and 0.1 mg/L IBA, ‘W4’—SH, 1.5 mg/L BA and 0.2 mg/L NAA. Means within each cultivar (*n* = 3) followed by different letters differ significantly (one-way ANOVA followed by Tukey’s multiple comparison test (*p* < 0.05)).

*V. vinifera* cvs.	Medium Variant *	DPPH Assay	ABTS Assay	FRAP Assay
Chardonnay	W1	0.71 ± 0.01 ^a^	0.33 ± 0.02 ^a^	1.69 ± 0.01 ^a^
	W2	0.49 ± 0.02 ^b^	0.35 ± 0.01 ^a^	0.82 ± 0.06 ^b^
	W3	0.01 ± 0.01 ^c^	0.33 ± 0.01 ^a^	1.06 ± 0.04 ^bc^
	W4	0.30 ± 0.06 ^d^	0.36 ± 0.03 ^a^	1.38 ± 0.16 ^ac^
Hibernal	W1	0.34 ± 0.01 ^a^	0.32 ± 0.03 ^a^	1.10 ± 0.08 ^a^
	W2	0.49 ± 0.14 ^a^	0.36 ± 0.02 ^ab^	1.14 ± 0.08 ^a^
	W3	0.30 ± 0.03 ^a^	0.40 ± 0.01 ^b^	0.73 ± 0.08 ^b^
	W4	0.42 ± 0.01 ^a^	0.37 ± 0.01 ^ab^	1.03 ± 0.06 ^ab^
Regent	W1	0.13 ± 0.03 ^a^	0.35 ± 0.01 ^a^	0.66 ± 0.12 ^a^
	W2	0.20 ± 0.02 ^ab^	0.35 ± 0.01 ^a^	0.73 ± 0.18 ^a^
	W3	0.12 ± 0.02 ^a^	0.35 ± 0.01 ^a^	0.94 ± 0.02 ^b^
	W4	0.27 ± 0.04 ^b^	0.34 ± 0.01 ^a^	0.86 ± 0.01 ^ab^
Marechal Foch	W1	0.82 ± 0.07 ^a^	0.34 ± 0.01 ^a^	1.47 ± 0.02 ^ab^
	W2	0.85 ± 0.02 ^a^	0.34 ± 0.02 ^a^	1.88 ± 0.04 ^a^
	W3	0.88 ± 0.02 ^a^	0.35 ± 0.05 ^a^	1.46 ± 0.05 ^ab^
	W4	0.77 ± 0.01 ^a^	0.35 ± 0.02 ^a^	1.08 ± 0.01 ^b^
Seyval Blanc	W1	0.78 ± 0.04 ^ab^	0.35 ± 0.01 ^a^	3.10 ± 0.05 ^a^
	W2	0.82 ± 0.01 ^ab^	0.36 ± 0.01 ^a^	2.88 ± 0.02 ^b^
	W3	0.86 ± 0.03 ^a^	0.41 ± 0.01 ^b^	1.07 ± 0.09 ^c^
	W4	0.69 ± 0.03 ^b^	0.40 ± 0.01 ^b^	0.69 ± 0.01 ^d^
Rondo	W1	0.37 ± 0.06 ^a^	0.27 ± 0.07 ^a^	1.13 ± 0.01 ^a^
	W2	0.27 ± 0.03 ^ab^	0.32 ± 0.04 ^a^	1.08 ± 0.02 ^ab^
	W3	0.19 ± 0.02 ^b^	0.36 ± 0.01 ^a^	0.91 ± 0.01 ^b^
	W4	0.32 ± 0.02 ^a^	0.37 ± 0.01 ^a^	0.96 ± 0.09 ^ab^

**Table 4:** The tyrosinase inhibition activity of extracts obtained from callus cultures of different *V. vinifera* cvs. Values are expressed as percentage inhibition (%). * ‘W1’—MS, 0.9 mg/L BA and 0.1 mg/L IBA, ‘W2’—MS, 1.5 mg/L BA and 0.2 mg/L NAA, ‘W3’—SH, 0.9 mg/L BA and 0.1 mg/L IBA, ‘W4’—SH, 1.5 mg/L BA and 0.2 mg/L NAA. Means within each cultivar (*n* = 3) followed by different letters differ significantly (one-way ANOVA followed by Tukey’s multiple comparison test (*p* < 0.05)).

*V. vinifera* cvs.	Medium Variant *	Tyrosinase Assay
Chardonnay	W1	65.06 ± 0.07 ^a^
	W2	63.60 ± 0.07 ^a^
	W3	63.58 ± 0.07 ^a^
	W4	60.19 ± 0.08 ^a^
Hibernal	W1	60.08 ± 0.08 ^a^
	W2	61.58 ± 0.07 ^a^
	W3	58.29 ± 0.09 ^a^
	W4	59.77 ± 0.09 ^a^
Regent	W1	55.62 ± 0.08 ^a^
	W2	58.62 ± 0.07 ^a^
	W3	57.52 ± 0.08 ^a^
	W4	61.86 ± 0.08 ^a^
Marechal Foch	W1	56.46 ± 0.09 ^a^
	W2	55.64 ± 0.09 ^a^
	W3	52.95 ± 0.10 ^a^
	W4	53.96 ± 0.09 ^a^
Seyval Blanc	W1	52.83 ± 0.08 ^a^
	W2	53.49 ± 0.09 ^a^
	W3	54.11 ± 0.09 ^a^
	W4	48.90 ± 0.09 ^a^
Rondo	W1	49.70 ± 0.10 ^a^
	W2	49.93 ± 0.09 ^a^
	W3	46.60 ± 0.09 ^a^
	W4	54.47 ± 0.09 ^a^

## Data Availability

The data are contained within the article.

## Supplementary Materials

The following supporting information can be downloaded at https://www.mdpi.com/article/10.3390/molecules31203610/s1, Table S1: Total content of the identified metabolites in extracts obtained from callus cultures of six *V. vinifera* cvs. grown under different culture conditions. Values are expressed as mg/g DW (mean ± SD). (nd*—not detected) (DP1*—monomeric stilbenoids, DP2*—dimeric stilbenoids) (‘W1’—MS, 0.9 mg/L BA and 0.1 mg/L IBA, ‘W2’—MS, 1.5 mg/L BA and 0.2 mg/L NAA, ‘W3’—SH, 0.9 mg/L BA and 0.1 mg/L IBA, ‘W4’—SH, 1.5 mg/L BA and 0.2 mg/L NAA). Means within each cultivar (n = 3) followed by different letters differ significantly (one-way ANOVA followed by Tukey’s multiple comparison test (*p* < 0.05).
