# Supplementary material for: Cultivar-Dependent Phytochemical Composition and Cosmetic Potential of Grapevine Callus Cultures
Source: Molecules. 2026 Oct 10;31(20):3610. doi: 10.3390/molecules31203610 (PMC13707480; doi:10.3390/molecules31203610)
Supplement: Supplementary file 1 [file molecules-31-03610-s001.zip › molecules-4581819-supplementary.pdf]

Table S1. Total content of the identified metabolites in extracts obtained from callus cultures of six *V. vinifera* cvs. grown under different culture conditions. Values are expressed as mg/g DW (mean ± SD). (nd* - not detected) (DP1* – monomeric stilbenoids, DP2* – dimeric stilbenoids) ('W1'—MS, 0.9 mg/L BA and 0.1 mg/L IBA, 'W2'—MS, 1.5 mg/L BA and 0.2 mg/L NAA, 'W3'—SH, 0.9 mg/L BA and 0.1 mg/L IBA, 'W4'—SH, 1.5 mg/L BA and 0.2 mg/L NAA). Means within each cultivar (n=3) followed by different letters differ significantly (one-way ANOVA followed by Tukey's multiple comparison test ($p < 0.05$).

V. vinifera cvs.	Medium Variant	Amino Acids	Flavan-3-ols	Stilbenoids DP1*	Stilbenoids DP2*
**Chardonnay**	W1	3.54±1.11[a]	132.67±14.40[a]	26.94±1.55[a]	0.28±0.04[a]
	W2	1.70±0.20[a]	86.61±18.53[b]	40.32±0.93[a]	0.50±0.04[a]
	W3	0.51±0.12[b]	52.17±12.35[b]	32.41±8.64[a]	87.46±28.23[b]
	W4	0.45±0.06[b]	103.04±28.24[b]	110.03±15.02[b]	314.94±44.76[c]
**Hibernal**	W1	4.48±1.35[a]	18.79±7.11[a]	158.61±21.12[a]	0.06±0.02[a]
	W2	1.19±0.01[a]	43.82±6.93[b]	84.55±29.25[a]	94.78±7.50[b]
	W3	1.24±0.67[a]	18.63±9.57[a]	200.48±43.36[a]	188.76±10.45[c]
	W4	0.81±0.16[a]	28.46±7.96[b]	108.26±40.25[a]	2.61±0.64[a]
**Regent**	W1	10.08±3.28[a]	22.56±0.28[a]	246.91±41.75[ab]	101.03±18.93[a]
	W2	10.31±1.17[a]	20.26±1.93[ab]	141.91±75.76[ab]	0.05±0.01[b]
	W3	1.86±0.10[b]	13.02±0.28[b]	93.05±31.70[a]	91.36±9.15[a]
	W4	1.95±0.17[b]	5.15±0.97[c]	253.86±44.33[b]	134.64±28.77[a]
**Marechal Foch**	W1	3.19±0.49[a]	101.69±7.59[a]	194.38±9.05[a]	118.62±8.55[a]
	W2	1.75±0.47[b]	98.02±33.81[a]	8.72±1.23[b]	0.30±0.02[b]
	W3	1.99±0.35[b]	31.56±6.95[b]	81.08±15.76[c]	231.61±18.26[c]
	W4	1.32±0.23[b]	2.31±2.12[b]	3.04±2.45[b]	nd*
**Seyval Blanc**	W1	10.87±0.96[a]	2.68±0.50[a]	57.92±2.30[a]	54.63±9.30[a]
	W2	7.14±0.31[a]	83.18±29.82[b]	34.79±2.08[a]	77.16±3.73[a]
	W3	1.27±0.30[a]	0.64±0.11[a]	11.07±1.76[a]	86.04±31.54[a]
	W4	1.60±0.14[a]	136.52±18.59[b]	99.39±7.94[a]	59.38±2.09[a]
**Rondo**	W1	4.41±1.08[a]	20.76±3.83[a]	8.26±0.69[ab]	33.80±1.56[a]
	W2	2.90±0.57[b]	22.17±2.09[a]	2.13±0.25[a]	55.60±3.12[b]
	W3	1.25±0.42[a]	24.80±2.21[a]	13.29±0.64[b]	26.84±0.13[ac]
	W4	0.87±0.01[a]	nd*	0.29±0.19[a]	0.17±0.11[c]
